# Quantifying Mitochondrial Dynamics in Patient Fibroblasts with Multiple Developmental Defects and Mitochondrial Disorders

**DOI:** 10.3390/ijms22126263

**Published:** 2021-06-10

**Authors:** Ajibola B. Bakare, Julienne Daniel, Joshua Stabach, Anapaula Rojas, Austin Bell, Brooke Henry, Shilpa Iyer

**Affiliations:** Department of Biological Sciences, J. William Fulbright College of Arts and Sciences, University of Arkansas, Fayetteville, AR 72701, USA; abbakare@uark.edu (A.B.B.); jgd003@uark.edu (J.D.); josh.stabach@gmail.com (J.S.); ar038@uark.edu (A.R.); awb006@uark.edu (A.B.); bah021@uark.edu (B.H.)

**Keywords:** mitochondrial dynamics, mitochondrial morphology, mitochondrial disorders, mitochondrial membrane potential, mitochondrial fission, mitochondrial fusion

## Abstract

Mitochondria are dynamic organelles that undergo rounds of fission and fusion and exhibit a wide range of morphologies that contribute to the regulation of different signaling pathways and various cellular functions. It is important to understand the differences between mitochondrial structure in health and disease so that therapies can be developed to maintain the homeostatic balance of mitochondrial dynamics. Mitochondrial disorders are multisystemic and characterized by complex and variable clinical pathologies. The dynamics of mitochondria in mitochondrial disorders is thus worthy of investigation. Therefore, in this study, we performed a comprehensive analysis of mitochondrial dynamics in ten patient-derived fibroblasts containing different mutations and deletions associated with various mitochondrial disorders. Our results suggest that the most predominant morphological signature for mitochondria in the diseased state is fragmentation, with eight out of the ten cell lines exhibiting characteristics consistent with fragmented mitochondria. To our knowledge, this is the first comprehensive study that quantifies mitochondrial dynamics in cell lines with a wide array of developmental and mitochondrial disorders. A more thorough analysis of the correlations between mitochondrial dynamics, mitochondrial genome perturbations, and bioenergetic dysfunction will aid in identifying unique morphological signatures of various mitochondrial disorders in the future.

## 1. Introduction

Mitochondria are highly dynamic organelles responsible for a host of cellular functions that include regulation of oxidative metabolism, control of apoptosis, and generation of signaling metabolites, with the production of adenosine triphosphate (ATP) being the most prominent of functions [1]. In live cells, work has shown that ATP production is regulated by remodeling of the mitochondria [2]. In healthy cells, mitochondrial structure differs from cell to cell, with the difference in morphology dictated by the function of the specific cell [2]. Mitochondria undergo rounds of fission and fusion to replenish the content of the organelle and to maintain structural integrity to continue to perform their function [3,4]. Alterations in mitochondrial morphology can cause bioenergetic defects and underlie a heterogeneous group of human diseases, including myopathies [5], neurodegeneration [6,7], cancer [8], diabetes mellitus [9,10], and a host of other disorders [3]. Owing to the involvement of mitochondrial dynamics in regulating cellular functions, studies have focused on understanding the relationship between the morphology and function of the mitochondria in health and disease.

Much is still unknown about the role of mitochondrial dynamics in human diseases. However, it has been suggested that in a diseased state, mitochondrial fission and fusion may be modulated as a compensatory mechanism to maintain the pool of healthy mitochondria within cells [4,11,12,13]. Mitochondrial fusion allows for the replenishment of depleted cellular resources, such as lipids and proteins between organelles, while fission generates new organelles and is involved in mitochondrial quality control [14]. While there have been reports implicating mitochondrial dynamics in neurodegenerative disorders and cardiovascular diseases [15,16], not much is known about the involvement of mitochondrial dynamics in mitochondrial disorders that result from perturbations in the mitochondrial genome.

In this study, we used the mitochondrial network morphology analysis (MiNA) tool [17], an ImageJ macro, to analyze the mitochondrial structure of fluorescently labeled human fibroblast cell lines. Live cells stained with Mitotracker Red CM-H_2_Xros (MTR), a dye that localizes in actively respiring mitochondria, were analyzed to quantify mitochondrial morphology in health and disease. We analyzed one control healthy fibroblast and 10 diseased fibroblast cell lines carrying various mitochondrial DNA (mtDNA) mutations and deletions. The diseased cell lines carry some of the most prevalent mutations and deletions involved in mitochondrial disorders, such as: Leigh syndrome (LS), Mitochondrial Encephalopathy, Lactic acidosis, Stroke-like episodes (MELAS), Kearns–Sayre Syndrome (KSS), and Pearson Syndrome (PS) [18]. We analyzed mitochondrial morphologies in these cell lines under coupled and uncoupled respiration conditions, wherein trifluoromethoxy carbonyl cyanide phenylhydrazone (FCCP), a mitochondrial uncoupler, was used to induce maximal respiration. FCCP treatment promotes mitochondrial depolarization, subsequently resulting in the fragmentation of mitochondrial networks [19]. Our results suggest that mitochondria from diseased fibroblast cells exhibit various remodeling of their mitochondria to regulate bioenergetic efficiency and energy expenditure, with the most prevalent morphological adaptation being fragmentation in the diseased cell lines. Furthermore, we noticed that the diseased cell lines tend to have fewer actively respiring mitochondria, suggesting reduced mitochondrial mass in the diseased state. Treatment with FCCP resulted in fragmentation in both healthy and diseased cell lines. By observing mitochondrial morphology in the presence and absence of FCCP, our work provides a better understanding of how healthy and diseased cells adapt to changes in ATP demand and supply. To our knowledge, this is the first comprehensive study that quantifies mitochondrial dynamics of cell lines with a wide array of developmental and mitochondrial disorders. These results could give us a broader view on how mitochondrial genome perturbations in the form of point mutations and deletions could contribute to altered mitochondrial dynamics and lead to bioenergetic dysfunction in mitochondrial disorders.

## 2. Results

### 2.1. Clinical Information and Cell Line Characteristics

The patient fibroblast cell lines were obtained from ten different patients with varying developmental and mitochondrial disorders. Three of the patient cell lines carry point mutations in the MTATP6 subunits (SBG1, SGB2: m.8993T>G, SBG3: m.9185T>C) of complex V (ATP synthase). Two of the patient cell lines have mtDNA mutations affecting the MTND3 (SBG4: m.10158T>C) and MTND5 (SBG5: m.12706T>C) subunits of complex I of the electron transport chain. These mutations affecting complex I and V have been implicated in Leigh syndrome (LS) [18,20,21,22,23]. LS is a classic mitochondrial disorder that affects mental and motor activity, where disease severity and developmental defects are tied to specific mutations and mutant load [18,24,25]. Fibroblast cell lines were also derived from patients with MELAS, with one line having the tRNA mutation (SBG6: m.3243A>G), while the other the SBG7: m.14739G>A mutation [26,27,28]. MELAS is a condition that affects many of the body’s systems, particularly the brain and the muscles with early symptoms in patients including muscle weakness and pain, recurrent headaches, loss of appetite, and seizures [26,27,28]. The remaining three fibroblast cell lines have mtDNA deletions and exhibit clinical symptoms associated with KSS [29,30] and PS [31]. KSS is a condition that affects many parts of the body with patients exhibiting progressive external ophthalmoplegia, ptosis, pigmentary retinopathy, cardiac conduction defects, ataxia, or abnormally high levels of protein in the fluid that surrounds and protects the brain and spinal cord (the cerebrospinal fluid or CSF) [29,30]. PS usually begins in infancy with half of the children dying in infancy or early childhood due to severe lactic acidosis or liver failure, with those who survive developing signs and symptoms related to KSS [31]. The three cell lines exhibited the following clinical symptoms and specific deletions: SBG8: 10676∆14868; SBG9: 7342∆9916; and SBG10: 10167∆15568 in the mitochondrial genome. The healthy control fibroblast cell line BJ was obtained from the American Type Culture Collection (Manassas, VA, USA). All patient fibroblast cell lines were obtained under approved IRB protocols at the source. We cultured all fibroblast cell lines at passage 8, to minimize variability in our analysis. In general, we observed differences in cellular proliferation rates with the healthy control BJ fibroblasts exhibiting a ~45 h doubling time, while the diseased lines exhibited a significantly longer (range of 50–100 h) doubling time. The significantly longer doubling time could be attributed to the presence of the mtDNA mutations and deletions that also correlate with reduced bioenergetics.

### 2.2. Descriptors of Mitochondrial Morphology 

**Figure 1:**
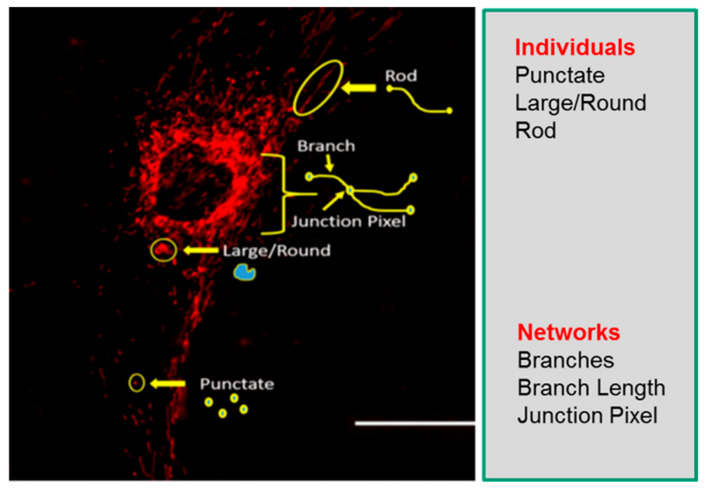
Mitochondrial morphology descriptors. The mitochondria morphology is classified as individuals or networks. Individuals consist of structures without a junction pixel, while networks contain branches with one or more junction pixels. Scale bar = 100 μm.

Understanding mitochondrial morphology is important in determining the health status of the cell, with descriptors such as tubular, fragmented, and hyperfused [32] often used to characterize mitochondrial morphology. These descriptors are important in identifying different mitochondrial morphologies in healthy and diseased cells and are also invaluable in making predictions on mitochondrial dynamics as they relate to cellular health. In our study, we have used the Mitochondrial Network Analysis (MiNA) toolset, a relatively simple pair of macros making use of existing ImageJ plugins, allowing for semiautomated analysis of mitochondrial morphologies in cultured mammalian cells [17]. MiNA combines rods, punctate, and large/round shaped morphology in a group as individuals, while branched morphologies are categorized as networks (Figure 1). Previous studies have indicated fragmented mitochondria to be the predominant morphology observed in mitochondrial dysfunctions [33,34], while fused mitochondria are associated with cell survival mechanisms [35,36]. It is therefore imperative that we identify these different morphologies to further delineate their contributions to cellular health and disease in the context of mitochondrial disorders.

### 2.3. Healthy Fibroblast Cell Lines Undergo Mitochondrial Fission When Substrate Is Abundant

**Figure 2:**
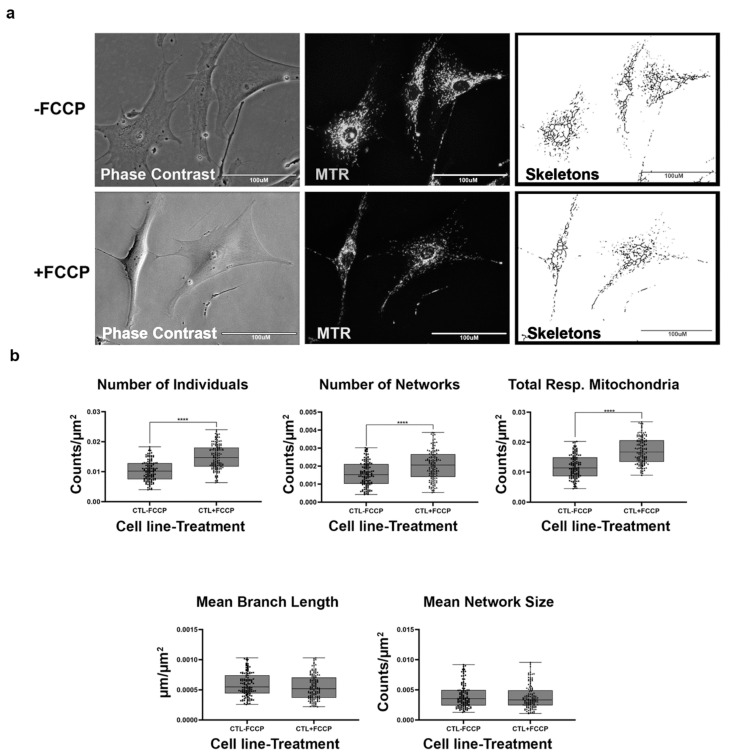
Mitochondrial morphology of healthy BJ fibroblast in the absence and presence of FCCP. (**a**) Representative images of fibroblast cell lines stained with Mitotracker Red CM-H2Xros (MTR), a dye that localizes to actively respiring mitochondria. The top images are phase contrast, RFP, and skeletonized images of healthy control cell lines without FCCP treatment. The bottom images are phase contrast, RFP, and skeletonized images of healthy control cell lines with FCCP treatment. (**b**) MiNA descriptors showing differences in mitochondrial morphology in untreated and FCCP treated groups. FCCP treatment resulted in mitochondrial fission and resulted in a significant increase in the number of individuals, networks, and total respiring mitochondria. The mean branch length and mean network size are lower after FCCP treatment, albeit not statistically significant. All data are representative of 10–14 images taken from five independent dishes per treatment group. The bars represent minimum and maximal values, and each black dot represents different data points. **** *p* < 0.0001. Scale bar = 100 μm.

Mitochondrial morphology in fibroblast cell lines was determined with the aid of live-cell fluorescent microscopy. To streamline our procedures, we first conducted an analysis with healthy BJ fibroblasts which are expected to exhibit various morphologies balanced between network and fragmented mitochondria (Figure 2a). In addition, we also treated the fibroblasts with FCCP to promote mitochondrial fragmentation. When the mitochondria undergo fission, the fragmentation of networks occurs. Conversely, mitochondrial fusion results in the formation of larger networks of mitochondria. The predictions for changes to be observed in mitochondrial morphology during fission and fusion events are detailed in Table 1. We predicted that healthy cells should be able to rapidly remodel their mitochondria to maintain energy homeostasis. This is evident by an increase in the number of individuals and networked mitochondria (Table 1) and a subsequent decrease in the mean branch lengths and network size. Our results support this prediction, as we recorded a 45% and 32% increase in the number of individuals and number of networks (Figure 2b). Total respiring mitochondria, which is the sum of individual and networked mitochondria, also increased by 46% in the FCCP treatment group. Although not statistically significant, mean branch length and mean network size with FCCP treatment were 7% and 5% lower, respectively. Using the MiNA macros, we were able to faithfully observe the dynamic nature of mitochondria in BJ healthy fibroblast cell lines.

### 2.4. Fibroblasts with Mutations Impacting Complex V Have Smaller, Fragmented Mitochondria

**Figure 3:**
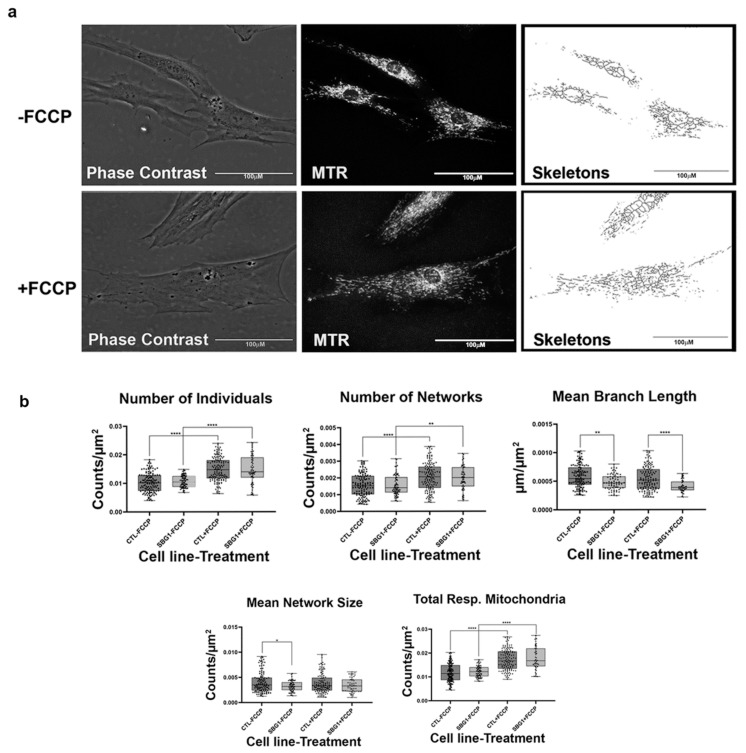
Mitochondrial morphology of SBG1 fibroblast in the absence and presence of FCCP. (**a**) Representative images of SBG1 fibroblast cell lines stained with MTR. The top images are phase contrast, RFP, and skeletonized images of SBG1 fibroblasts without FCCP treatment. The bottom images are phase contrast, RFP, and skeletonized images of SBG1 fibroblasts with FCCP treatment. (**b**) Although SBG1 cell lines have a comparable number of individual and networked mitochondria to control, the mitochondria in these lines are smaller. This is evident in the significant decrease in mean branch length and mean network size relative to control under basal conditions, without FCCP treatment. Treatment with FCCP resulted in mitochondrial fission, with SBG1 showing a response to FCCP similar to the healthy control. All data are representative of 10–14 images taken from three independent dishes per treatment group. The bars represent minimum and maximal values, and each black dot represents different data points. **** *p* < 0.0001, ** *p* < 0.01, * *p* < 0.05. Scale bar = 100 μm.

**Figure 4:**
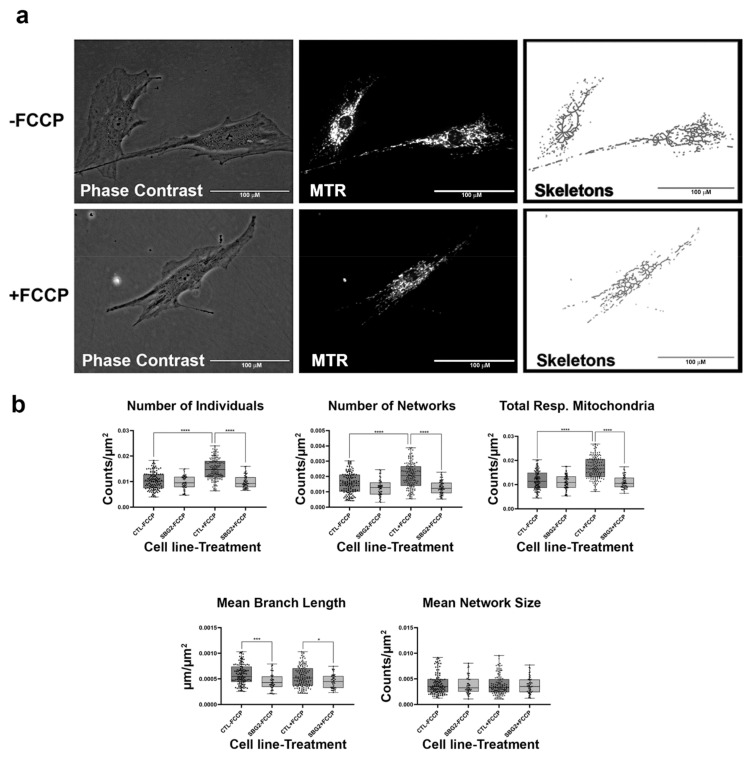
Mitochondrial morphology of SBG2 fibroblast in the absence and presence of FCCP. (**a**) Representative images of SBG2 fibroblast cell lines stained with MTR. The top images are phase contrast, RFP, and skeletonized images of SBG2 fibroblasts without FCCP treatment. The bottom images are phase contrast, RFP, and skeletonized images of SBG2 fibroblasts with FCCP treatment. (**b**) SBG2 fibroblasts have a slightly lower number of individual and networked mitochondria compared to control BJ fibroblasts. The significantly lower mean branch length relative to control fibroblasts under basal conditions further suggests fragmentation. SBG2 fibroblasts are not responsive to FCCP treatment. All data are representative of 10–14 images taken from three independent dishes per treatment group. The bars represent minimum and maximal values, and each black dot represents different data points. **** *p* < 0.0001, *** *p* < 0.001, * *p* < 0.05. Scale bar = 100 μm.

**Figure 5:**
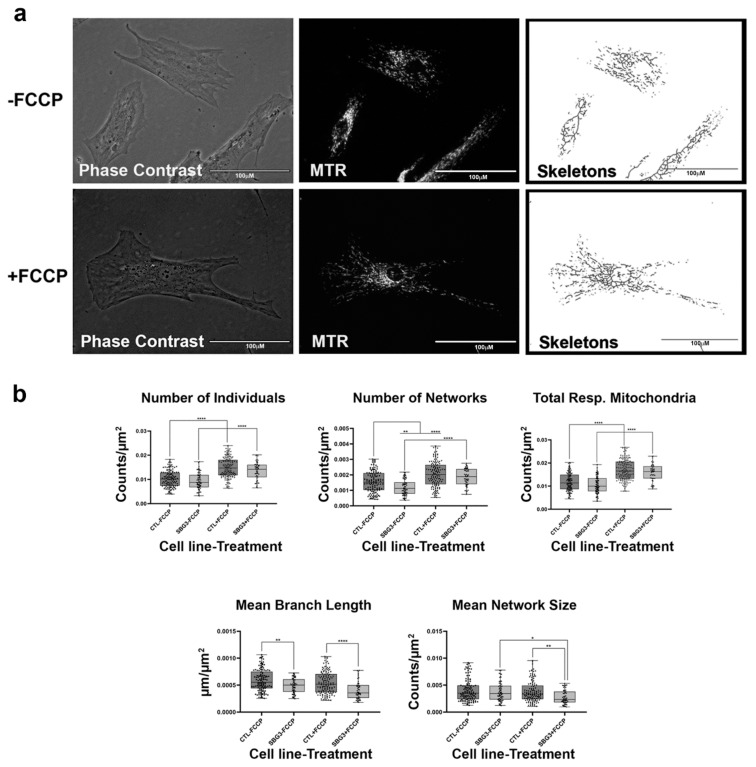
Mitochondrial morphology of SBG3 fibroblast in the absence and presence of FCCP. (**a**) Representative images of SBG3 fibroblasts stained with MTR. The top images are phase contrast, RFP, and skeletonized images of SBG3 fibroblasts without FCCP treatment. The bottom images are phase contrast, RFP, and skeletonized images of SBG3 fibroblasts with FCCP treatment. (**b**) While the number of individuals in SBG3 fibroblasts is comparable to the healthy control BJ fibroblasts, the number of networks is significantly lower. This shows that SBG3 fibroblasts have fewer networks, with significantly lower mean branch length further suggesting that the networks have smaller branches. Together, this demonstrates a more fragmented morphology in SBG3 fibroblasts with FCCP treatment resulting in more fragmentation, as expected. All data are representative of 10–14 images taken from three independent dishes per treatment group. The bars represent minimum and maximal values, and each black dot represents different data points. **** *p* < 0.0001, ** *p* < 0.01, * *p* < 0.05. Scale bar = 100 μm.

Having confirmed that the MiNA toolset would detect changes in mitochondrial dynamics in healthy control BJ fibroblasts, we proceeded to analyze differences between healthy control and diseased cell lines carrying mutations in subunits of the Complex V-ATP synthase. We analyzed three patient fibroblast cell lines (SBG1 and SBG2: m.8993T>G; SBG3: m.9185T>C) carrying mutations that are most prevalent in patients with LS [37,38]. We analyzed mitochondrial morphology in the diseased fibroblasts under basal conditions and after FCCP treatment. The SBG1 cell line exhibited trends towards slightly more fragmented mitochondria than the healthy control (Figure 3a). This is evident by the 4% and 1% increase in the number of individuals and networks, respectively, and the subsequent decrease in mean branch length and mean network size (Figure 3b). However, the SBG2 cell line with the same mutation had a different mitochondrial profile (Figure 4a,b). Both SBG2 and SBG3 (Figure 5a,b) fibroblasts had total respiring mitochondria counts that were lower than those of the control BJ fibroblast, suggesting fewer actively respiring mitochondria. Although there was a trend towards less individual and networked mitochondria in SBG2 and SBG3 fibroblasts relative to the control fibroblasts, all diseased lines had lower mean branch length and mean network size, supporting the presence of more fragmented morphology in the diseased fibroblasts. Treatment with FCCP resulted in fragmentation in both SBG1 and SBG3 fibroblasts, with both of these cell lines following the same trend as seen in the healthy control BJ fibroblast. However, SBG2 fibroblast was not as responsive to FCCP treatment, and there was no marked increase in either number of individual or networked mitochondria (Figure 4b). Furthermore, the mean branch length and mean network size exhibited similar values between the untreated and FCCP treatment groups. Together, these results indicate that the presence of the same mutations could result in different compensatory responses by the mitochondria, and variable bioenergetic demand within the fibroblasts could result in different mitochondrial dynamics. 

### 2.5. Fibroblasts with MTND5 Mutation Exhibit Hyperfused Mitochondria

**Figure 6:**
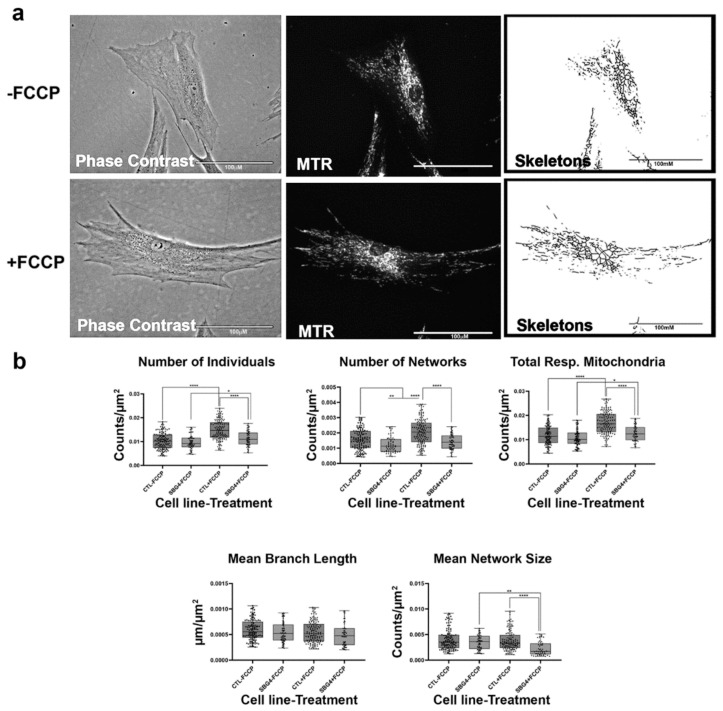
Mitochondrial morphology of SBG4 fibroblasts in the absence and presence of FCCP. (**a**) Representative images of SBG4 fibroblast cell lines stained with MTR. The top images are phase contrast, RFP, and skeletonized images of SBG4 fibroblasts without FCCP treatment. The bottom images are phase contrast, RFP, and skeletonized images of SBG4 fibroblasts with FCCP treatment. (**b**) Aside from having fewer networks, SBG4 fibroblasts are not significantly different from the control BJ fibroblasts. However, they are also responsive to FCCP treatment similar to the healthy control BJ fibroblasts. All data are representative of 10–14 images taken from three independent dishes per treatment group. The bars represent minimum and maximal values, and each black dot represents different data points. **** *p* < 0.0001, ** *p* < 0.01, * *p* < 0.05. Scale bar = 100 μm.

**Figure 7:**
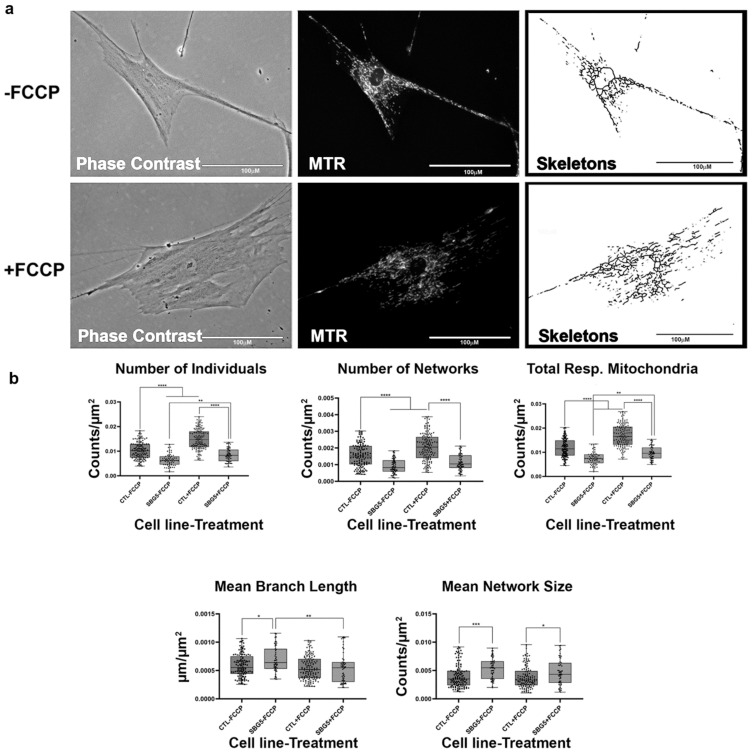
Mitochondrial morphology of SBG5 fibroblasts in the absence and presence of FCCP. (**a**) Representative images of SBG5 fibroblast cell lines stained with MTR. The top images are phase contrast, RFP, and skeletonized images of SBG5 fibroblasts without FCCP treatment. The bottom images are phase contrast, RFP, and skeletonized images of SBG5 fibroblasts with FCCP treatment. (**b**) The amount of actively respiring mitochondria in SBG5 fibroblasts is lower than those of the healthy control BJ fibroblasts. The significantly lower number of individuals, networks, and total respiring mitochondria in the SBG5 fibroblasts support this observation. The SBG5 fibroblasts also have hyperfused mitochondria, as they have longer branches and more branches within their networks. All data are representative of 10–14 images taken from three independent dishes per treatment group. The bars represent minimum and maximal values, and each black dot represents different data points. **** *p* < 0.0001, *** *p* < 0.001, ** *p* < 0.01, * *p* < 0.05. Scale bar = 100 μm.

We next wanted to determine how mutations in other complexes that impact mitochondrial function affect mitochondrial morphology. We assessed this using two patient fibroblast cell lines with mutations affecting subunits of complex I of the electron transport chain. The SBG4 fibroblast cell line has the m.10158T>C point mutation, while the SBG5 fibroblast has the m.12706T>C point mutation affecting MTND3 and MTND5 subunits, respectively. On average both SBG4 and SBG5 fibroblasts trended towards having fewer actively respiring mitochondria than the healthy control BJ fibroblast, as evidenced by the relatively lower total respiring mitochondria (Figure 6 and Figure 7). This difference is more pronounced in the SBG5 fibroblasts, which have a 37% decrease compared to the 9% decrease recorded for the SBG4 fibroblasts. As expected, FCCP treatment resulted in fragmented mitochondria in the SBG4 fibroblasts (Figure 6b). The SBG5 fibroblasts exhibit a hyperfused morphology (Figure 7b) under basal conditions with significantly elevated (*p* < 0.0001) mean branch length and mean network size compared to healthy control BJ fibroblasts. FCCP treatment reversed this phenotype, with mean branch length and mean network size within the SBG5 group decreasing by 21% and 14%, respectively, after FCCP treatment. This is consistent with mitochondria fragmentation by FCCP, breaking down the larger networks of mitochondria in this cell line. Unlike what we observed with the SBG1, SBG2, and SBG3 fibroblasts (with mutations impacting Complex V), SBG4 and SBG5 fibroblasts (with mutations impacting Complex I) appear to exhibit different morphologies. These differences could be attributed to the location of the subunits affected by each mutation. The mutation in SBG5 fibroblasts may affect the function of the mitochondria more than that of SBG4 fibroblasts, requiring remodeling of the mitochondria to maintain optimal function of the respiratory chain. 

### 2.6. Mitochondrial tRNA Mutations Result in Fewer Actively Respiring Mitochondria

**Figure 8:**
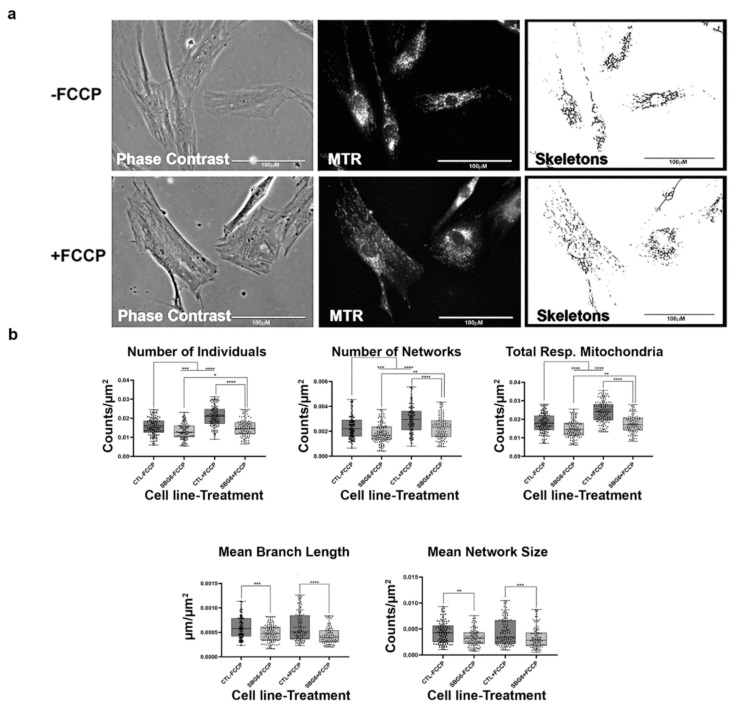
Mitochondrial morphology of SBG6 fibroblast in the absence and presence of FCCP. (**a**) Representative images of SBG6 fibroblast cell lines stained with MTR. The top images are phase contrast, RFP, and skeletonized images of SBG6 cell lines without FCCP treatment. The bottom images are those of the FCCP treatment group. (**b**) The SBG6 cell lines have significantly fewer networks of actively respiring mitochondria relative to the control cell lines. All data are representative of 10–14 images taken from three independent dishes per treatment group. The bars represent minimum and maximal values, and each black dot represents different data points. **** *p* < 0.0001, *** *p* < 0.001, ** *p* < 0.01, * *p* < 0.05. Scale bar = 100 μm.

**Figure 9:**
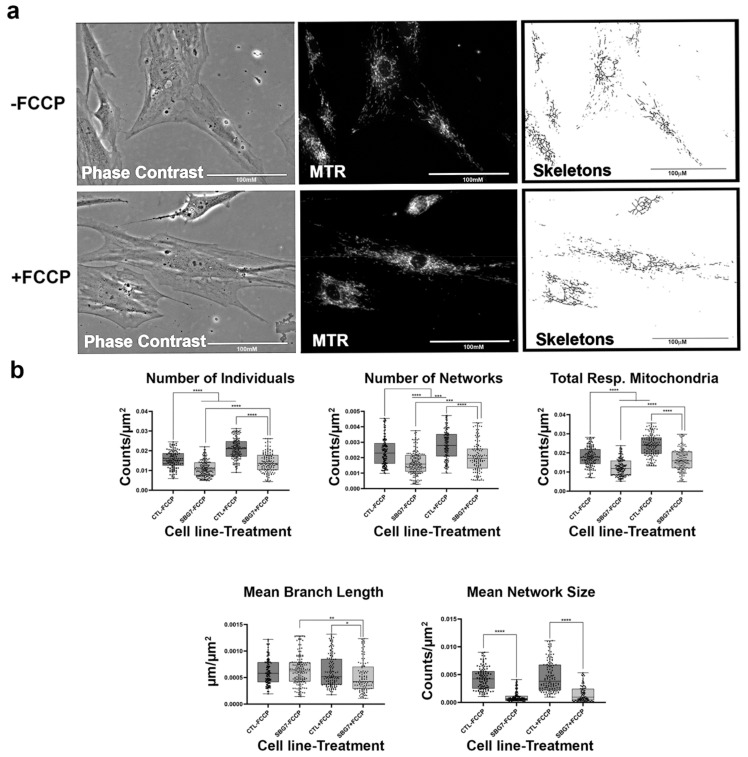
Mitochondrial morphology of SBG7 fibroblast in the absence and presence of FCCP. (**a**) Representative images of SBG7 fibroblast cell lines stained with MTR. The top images are phase contrast, RFP, and skeletonized images of SBG7 fibroblasts without FCCP treatment. The bottom images are phase contrast, RFP, and skeletonized images of SBG7 fibroblasts with FCCP treatment. (**b**) The SBG7 fibroblasts have significantly fewer networks of actively respiring mitochondria relative to the control BJ fibroblasts. FCCP treatment resulted in fragmented mitochondria in the SBG7 fibroblasts with mean network size trended towards an increase upon treatment. All data are representative of 10–14 images taken from three independent dishes per treatment group. The bars represent minimum and maximal values, and each black dot represents different data points. **** *p* < 0.0001, *** *p* < 0.001, ** *p* < 0.01, * *p* < 0.05. Scale bar = 100 μm.

We next examined mitochondria morphology in patient cell lines with tRNA mutations. One of the cell lines, SBG6, carries an m.3243A>G point mutation. This mutation is present in 80% of MELAS patients [39,40]. The other patient cell line, SBG7, carries the point mutation m.14739G>A. This mutation also affects the function of the tRNA by unknown mechanisms to cause mitochondrial pathology. Interactions between bases in tRNA are very important for maintaining the tertiary structure. These mutations alter the tertiary structure of the tRNA, which impairs mitochondrial translation machinery [40] and decreases overall mitochondrial respiration. Indeed, we observed a general decrease in mitochondrial respiration. Both SBG6 and SBG7 cell lines (Figure 8 and Figure 9) have a significantly lower (*p* < 0.001) number of individual, networked, and total respiring mitochondria and mean network size relative to the control cell lines. Similar to the healthy control, FCCP treatment resulted in fragmentation in both SBG6 and SBG7 cell lines. Interestingly, we see a significant increase of 51% in mean network size in the SBG7 cell line after FCCP treatment. It is not fully clear why the network size increased in SBG7 cell lines after FCCP treatment; perhaps this is because of the slightly longer branch length (5%) of SBG7 relative to the healthy control. 

### 2.7. Fibroblasts with mtDNA Deletions Exhibit Fragmented Mitochondria

**Figure 10:**
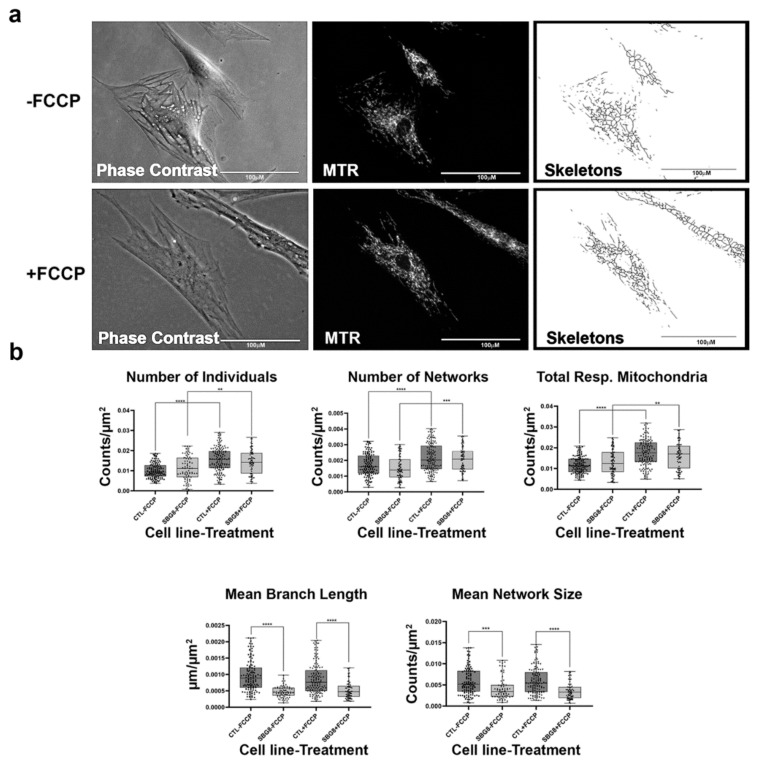
Mitochondrial morphology of SBG8 fibroblast in the absence and presence of FCCP. (**a**) Representative images of SBG8 fibroblast cell lines stained with MTR. The top images are phase contrast, RFP, and skeletonized images of SBG8 fibroblast cell lines without FCCP treatment. The bottom images are phase contrast, RFP, and skeletonized images of SBG8 fibroblasts with FCCP treatment. (**b**) SBG8 fibroblasts have significantly shorter branch lengths and fewer branches within their networks. All data are representative of 10–14 images taken from three independent dishes per treatment group. The bars represent minimum and maximal values, and each black dot represent different data points. **** *p* < 0.0001, *** *p* < 0.001, ** *p* < 0.01 Scale bar = 100 μm.

**Figure 11:**
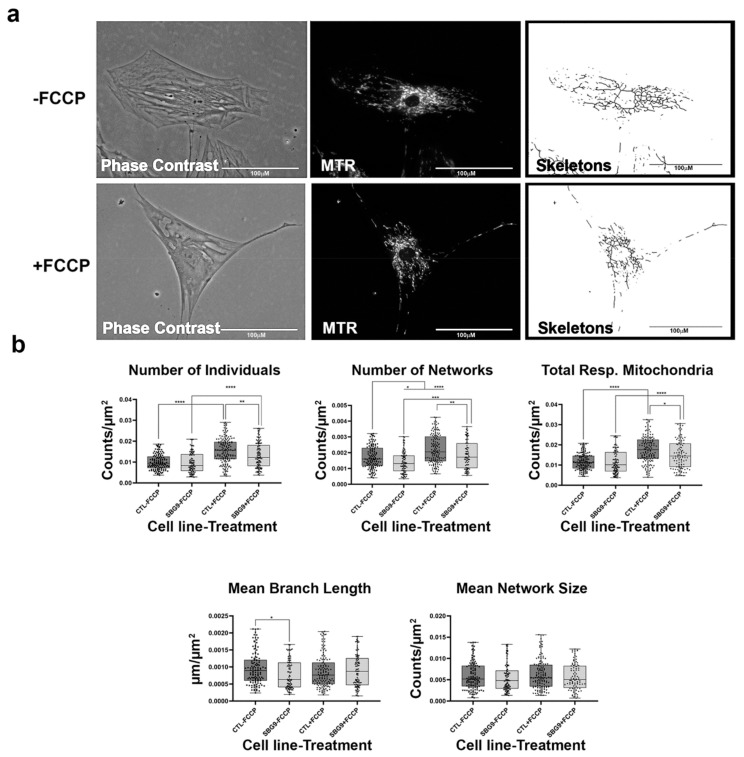
Mitochondrial morphology of SBG9 fibroblast in the absence and presence of FCCP. (**a**) Representative images of SBG9 fibroblast cell lines stained with MTR. The top images are phase contrast, RFP, and skeletonized images of SBG9 fibroblast cell lines without FCCP treatment. The bottom images are phase contrast, RFP, and skeletonized images of SBG9 fibroblasts with FCCP treatment. (**b**) The mean branch length is significantly lower in SBG9 fibroblast cell lines relative to the healthy BJ control fibroblasts. All data are representative of 10–14 images taken from three independent dishes per treatment group. The bars represent minimum and maximal values, and each black dot represents different data points. **** *p* < 0.0001, *** *p* < 0.001, ** *p* < 0.01, * *p* < 0.05. Scale bar = 100 μm.

**Figure 12:**
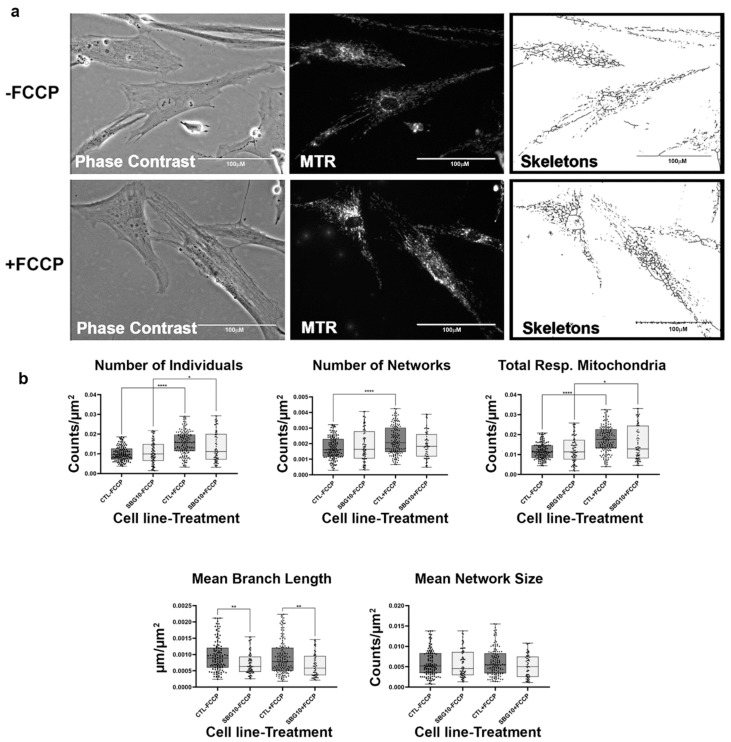
Mitochondrial morphology of SBG10 fibroblast in the absence and presence of FCCP. (**a**) Representative images of SBG10 fibroblast cell lines stained with MTR. The top images are phase contrast, RFP, and skeletonized images of SBG10 fibroblast cell lines without FCCP treatment. The bottom images are phase contrast, RFP, and skeletonized images of SBG10 fibroblasts with FCCP treatment. (**b**) The mean branch length is significantly lower in SBG10 cell lines relative to the healthy control. All data are representative of 10–14 images taken from three independent dishes per treatment group. The bars represent minimum and maximal values, and each black dot represents different data points. **** *p* < 0.0001, ** *p* < 0.01, * *p* < 0.05. Scale bar = 100 μm.

Finally, we examined the effects of mtDNA deletions on mitochondrial morphology. We used three different patient fibroblast cell lines with varying degrees of mtDNA deletions (SBG8 derived from a pediatric KSS patient with a 4.1 kb mtDNA deletion; SBG9 derived from a patient with PS with a 2.5 kb deletion; SBG10 derived from a patient with PS with a 5.4 kb deletion). Although not statistically significant, we observe a trend towards fission based on an increased presence of fragmented mitochondria in SBG8 and SBG10 fibroblasts, but not in SBG9 fibroblasts (Figure 10, Figure 11 and Figure 12). The relative increase in the number of individuals and total respiring mitochondria and subsequent decrease in the mean branch length of these cell lines compared to those of the healthy control BJ fibroblast follows the prediction for fragmented mitochondria (Table 1). The SBG9 (Figure 11) fibroblast, however, exhibited a profile similar to that of the healthy control BJ fibroblast cell lines, an observation attributed to differences in the deletion thus impacting mitochondrial dynamics. After FCCP treatment, all the diseased fibroblast cell lines became more fragmented with a significant increase in the number of individual, networked, and total respiring mitochondria (Figure 10, Figure 11 and Figure 12). 

### 2.8. Mitochondrial Membrane Potential Was Decreased in All Diseased Cell Lines

**Figure 13:**
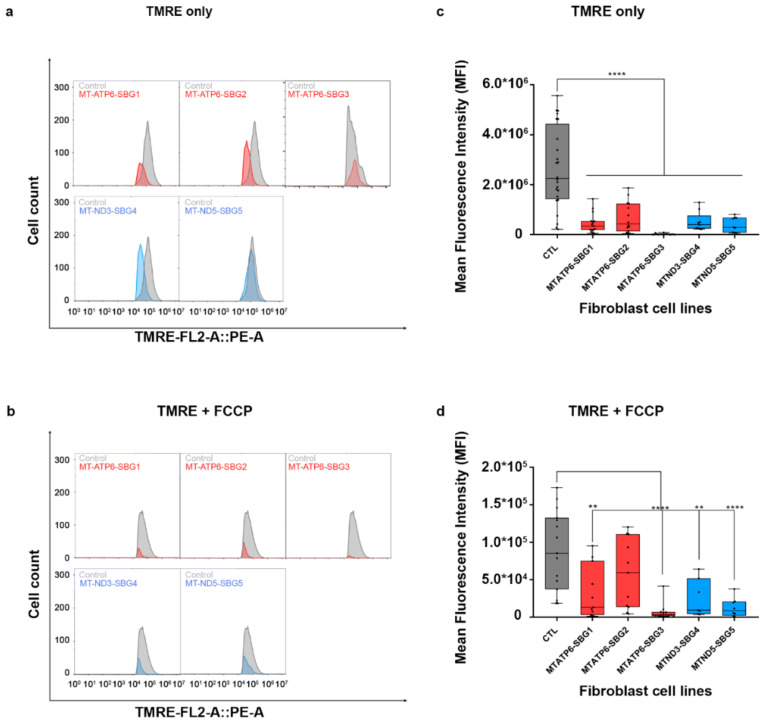
Mitochondrial membrane potential (MMP) analysis of BJ-FB and LS fibroblast cell lines. Using flow cytometry, along with membrane-potential sensitive dye (TMRE), MMP was evaluated. Representative flow cytometry histogram of five fibroblasts (SBG1–5 and Control BJ) (**a**) stained with TMRE only; (**b**) stained with TMRE after treatment with FCCP. Mean fluorescence intensity (MFI) was calculated based on three independent runs, which are shown for (Control BJ-FB in grey; SBG1-FB (MT-ATP6-T8993G), SBG2-FB (MT-ATP6- T8993G), and SBG3-FB (MT-ATP6-T9185C) in red; SBG4-FB (MT-ND3-T10158C) and SBG5-FB (MT-ND5-T12706C) in blue) all samples (**c**) stained with TMRE only; (**d**) stained with TMRE after treatment with FCCP. ** *p* < 0.01, **** *p* < 0.00001.

**Figure 14:**
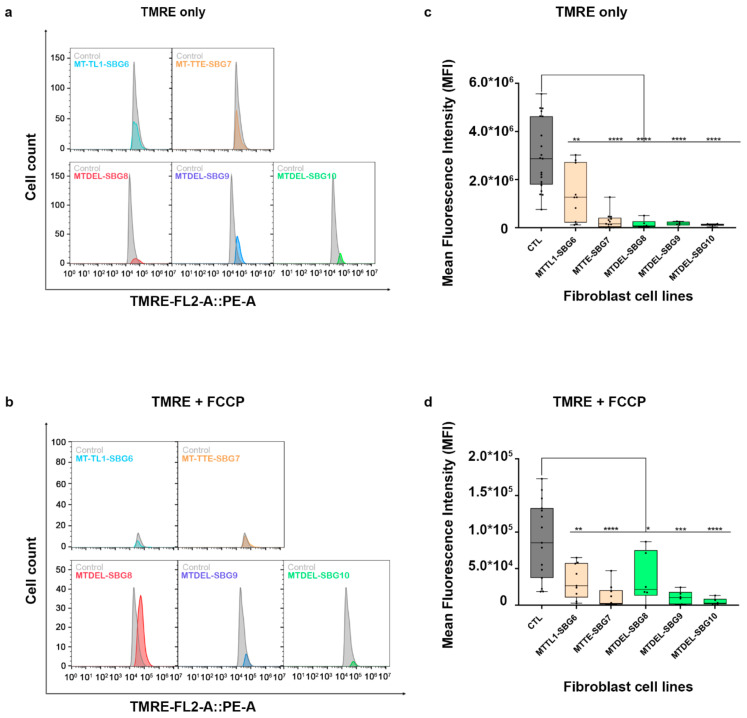
Mitochondrial membrane potential (MMP) analysis of BJ-FB diseased fibroblast cell lines. Using flow cytometry, along with membrane-potential sensitive dye (TMRE), MMP was evaluated. Representative flow cytometry histogram of five fibroblasts (SBG6–10 and Control BJ) (**a**) stained with TMRE only; (**b**) stained with TMRE after treatment with FCCP. Mean fluorescence intensity (MFI) was calculated based on three independent runs and are shown for (Control BJ-FB in grey; SBG6-FB (m.3243A>G) and SBG7-FB (m.14739G>A) in tan; SBG8-FB (10676∆14868), SBG9-FB (7342∆9916), and SBG10-FB (10167∆15568) in green) all samples (**c**) stained with TMRE only; (**d**) stained with TMRE after treatment with FCCP. * *p* < 0.05, ** *p* < 0.01, *** *p* < 0.001, **** *p* < 0.00001.

To investigate how the changes in mitochondrial dynamics affect mitochondrial function, we recorded mitochondrial membrane potential (MMP) in the diseased and healthy control fibroblast cell lines. We hypothesized that MMP would be perturbed in the diseased cell lines by different mechanisms. MMP generated by proton pumps at complex I, III, and IV is an essential component in the process of energy storage [41,42]. Flow cytometry methods have been used to measure MMP with tetramethylrhodamine, ethyl ester (TMRE) in live cells with no quenching effect [43]. TMRE is a positively charged dye that is attracted to the negative potential across the inner mitochondrial membrane and thus accumulates in functionally active mitochondria in live cells [44]. As active mitochondria maintain a net negative charge in the matrix, TMRE is sequestered in the matrix of these mitochondria. Depolarized or inactive mitochondria are not able to sequester TMRE as the MMP is compromised in these mitochondria. In our study, MMP was measured as mean fluorescent intensity (MFI) in all cell lines. Results indicate a significant (*p* < 0.0001) decrease in MMP by 83% in SBG1-FB (m.8993T>G), 74.13% in SBG2-FB (m.8993T>G), 99.23% in SBG3-FB (m.9185T>C), 80.47% in SBG4-FB (m.10158T>C), and 84.92% in SBG5-FB (m.12706T>C) compared to healthy control BJ-FB cells (Figure 13a,c). In the cell lines with tRNA mutations, MMP was lower (*p* < 0.001) by 53.91% in SBG6-FB (m.3243A>G) and 90.61% in SBG7-FB (m.14739G>A) relative to healthy control BJ-FB cells (Figure 14a,c). As expected, the cell lines with mtDNA deletions also showed a significant (*p* < 0.0001) decrease in MMP by 94.86% in SBG8-FB (10676∆14868), 94.50% in SBG9-FB (7342∆9916), and 96.12% in SBG10-FB (10167∆15568) compared to healthy control BJ-FB cells (Figure 14a,c). Although the uncoupler cyanide *p*-trifluoromethoxyphenylhydrazone (FCCP) decreased the MMP significantly for most of the diseased cell lines relative to control BJ-FB (*p* < 0.05), SBG2 was not significantly different from the healthy BJ-FB control (Figure 13b,d and Figure 14b,d).

## 3. Discussion

**Figure 15:**
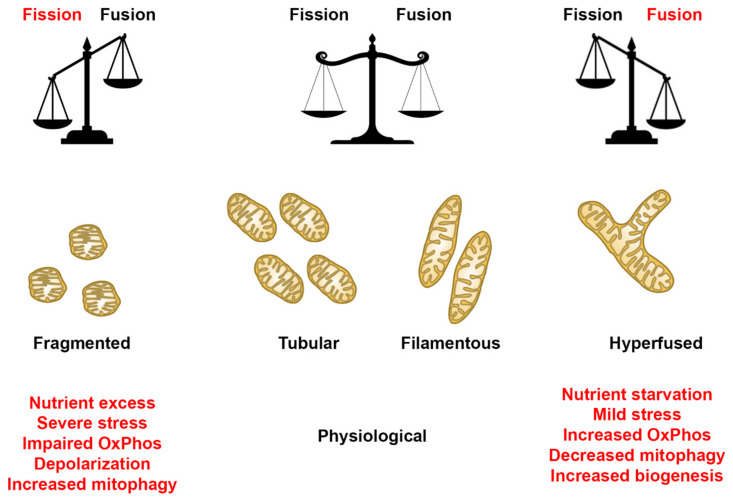
Mitochondrial morphology summary for fibroblast cell lines. Comparison of mitochondrial morphology of diseased and healthy fibroblast cell lines under basal conditions. Results suggest that diseased cell lines tend to have fewer respiring mitochondria with small branches and fragmented networks. In some cases, however, as seen in SBG5 and SBG7 fibroblasts, hyperfusion serves as a compensatory mechanism.

Mitochondria are dynamic organelles that perform a plethora of cellular processes, including ATP production, regulation of ions, apoptosis, and cellular signaling, processes that depend on tight regulation of mitochondrial shapes and ultrastructure [1,45]. Recent studies have provided evidence that highlights the importance of mitochondrial dynamics in various diseases [7,8,15,46]. Additionally, studies have demonstrated the mitochondria’s ability to display various net morphologies, depending on the cell type and/or metabolic state [47]. Under physiological conditions, a balance between fission and fusion helps facilitate normal mitochondrial function (Figure 13). However, in a diseased/stressed state, our findings indicate that the mitochondrial dynamics can be predisposed to an overt fission or fusion rate, depending upon the mitochondrial mutation. Under these conditions, the mitochondria response that upregulates fission or fusion mechanisms results in a fragmented or hyperfused morphology, respectively (Figure 15). In a fragmented state, mitochondria are more spherical, rounded individuals, while hyperfused mitochondria morph into large networks (Figure 15). The degree to which mitochondria in a cell undergo such alterations could vary depending on various factors. Therefore, understanding the role of mitochondrial dynamics in various mitochondrial disorders could be instrumental in filling the knowledge gap that exists in the context of the relationship between mtDNA mutations/deletions and mitochondrial morphology.

Previous studies have focused on mitochondrial dynamics in various diseases [15,17,47,48,49] and have provided key insights into its importance in the context of understanding changes that occur during pathological states. However, this is the first study that provides a comprehensive view of mitochondrial morphology and dynamics across different mitochondrial disorders based on analysis of ten fibroblasts derived from patients diagnosed with LS, MELAS, KSS, and PS. To ensure that the MiNA tool that quantifies mitochondrial morphology based on networked and unbranched mitochondria [17] was effective in our analysis, we treated healthy control cell lines with FCCP. FCCP is an uncoupler that stimulates maximal respiratory capacity and stresses the mitochondria, consequently causing mitochondrial network fragmentation [19,50]. Treatment of the control fibroblast cell lines with FCCP resulted in a significant increase in the number of individuals and networks (Figure 2b). This increase coupled with a 7% and 5% decrease in mean branch length and mean network size, respectively, confirms mitochondrial fragmentation induced by FCCP. Total respiring mitochondria, the sum of the number of individuals and networks, also increased significantly, a result that is consistent with previous studies that have suggested that fragmentation is advantageous for uncoupled respiration [19,51].

After confirming that MiNA is sensitive and can be used to characterize mitochondrial morphology, we used it to quantify the morphology of the diseased fibroblast cell lines containing specific mutations or deletions. Across all ten cell lines with different mitochondrial disorders, about half have the same mitochondrial morphology profile when compared to the healthy BJ control fibroblast (Table 2). In these cell lines (SBG2, SBG3, SBG4, SBG6, and SBG9), there is a trend towards smaller fragmented mitochondria, with a relatively lower number of individuals, networks, mean branch length, mean network size, and total respiring mitochondria. The shorter branch length and fewer network branches suggest that the networks of mitochondria in these cell lines are smaller than observed in the healthy control BJ fibroblasts. Even in the other fibroblast cell lines (SBG1 and SBG8) that have slightly different profiles, we still observe a significant decrease in the mean branch length. These results suggest that the fibroblasts that contain specific mtDNA mutations exhibit mitochondrial fragmentation, with only SBG5 and SBG7 fibroblast cell lines deviating from this morphology profile. In the SBG5 cell lines, the mean branch length and mean network size are significantly greater than the healthy control with the potential for *MTND5* mutation in this cell line favoring mitochondrial fusion. Although the SBG7 fibroblast cell line also displays an elevated mean branch length, there is no subsequent increase in the mean network size. Therefore, we surmise that the SBG5 fibroblast cell lines exhibit an extreme form of fusion compared to the SBG7 fibroblast cell line, with SBG5 fibroblast cell lines exhibiting hyperfused morphology.

When the cell lines were treated with FCCP, we noted that some of the diseased cell lines exhibited the same response to FCCP as the healthy control cell lines, while others did not. We also observed similar trends with basal (oxygen consumption rate) and maximal respiration values using a Seahorse Flux Analyzer. The basal respiration values were significantly reduced for 8 out of the 10 fibroblasts, while only SBG1 exhibited a slight increase and SBG10 exhibited no change when compared with healthy control BJ-FB. Upon addition of FCCP, the maximal respiration values were significantly increased for 5 out of the 10 fibroblasts (SBG1, SBG3, SBG4, SBG7, SBG8) and reduced for SBG2, SBG5, and SBG9, while SBG6 and SBG10 exhibited no change when compared with healthy control BJ-FB. Based on ongoing studies in our laboratory, results indicate that variable respiration rates are dependent on specific mtDNA mutations and deletions in the different disease fibroblasts. (Bakare and Iyer, Personal communication). A common view of mitochondrial disorders harboring mtDNA mutations or deletions is that there is a direct correlation between disease severity and outcomes to the degree of heteroplasmy. Contrary to this view, our preliminary results based on comprehensive next generation sequencing analysis indicate varying heteroplasmy levels (between 25 and 98%) in the different fibroblasts. These results are consistent with two recent large cohort studies on the phenotypic spectrum of mitochondrial disorders not correlating with the degree of mutation heteroplasmy [52,53]. Two of the fibroblast cell lines with mtDNA deletion, SBG8, and SBG9 responded differently to FCCP. In these cell lines, FCCP exposure resulted in fusion, with a slight increase of 12% and 15% in mean branch length recorded for SBG8 and SBG9 cell lines, respectively. Mitochondrial fusion provides an advantage to cells under high-energy demands [54] by promoting biogenesis (Figure 15). Given that the SBG8 and SBG9 fibroblasts already have large deletions in mtDNA, it is possible that when FCCP is added, instead of undergoing fission as expected, the mitochondria in these cells might be compensating by fusing to meet the energetic demands.

Our findings support previous results where fragmented mitochondria are the predominant morphology in the diseased state [48,54,55,56]. In addition, the mitochondrial functional analysis based on mitochondrial membrane potential (MMP) measurements demonstrates the differences that exist between diseased and healthy states. This is in line with numerous studies that have demonstrated that changes in MMP and mitochondrial morphological alterations often occur in parallel [57,58,59]. Our study for the first time has characterized mitochondrial morphology across ten different patient cell lines with varying mitochondrial disorders. Our results describe a strong relationship between mtDNA genome perturbations (mutations/deletions) and alterations in mitochondrial morphology in these patient fibroblast cell lines. While analysis using MiNA was able to provide us with the necessary information required for this comprehensive comparative study, there are still some limitations to this tool. For example, MiNA groups round, punctate, and rods together as individuals. It would be beneficial to distinguish between fragmentations that result in the formation of more round, punctate mitochondria as opposed to rods, as this would help with better delineation of the mitochondrial dynamics in diseased cell lines. In addition, comparative analysis has been conducted with one healthy control fibroblast (BJ-FB) cell line. Its use in these studies was based on its neonatal origin, and it was used in the context of comparing with disease fibroblasts related to ‘pediatric’ mitochondrial disorders. Future studies will be conducted with multiple control fibroblasts that will allow us to address gender and age as well as allow for comparison of mitochondrial dynamics across lifespan. Nevertheless, this quantitative analysis allowed us to identify differences in mitochondrial dynamics in fibroblasts that exhibit candidate mtDNA mutations or deletions. In the long term, we hope that a thorough evaluation of mitochondrial dynamics along with correlations with bioenergetic and genetic analyses will aid in identifying the ‘signature’ of complex mitochondrial disorders and contribute to the development of effective therapies.

## 4. Materials and Methods

### 4.1. Fibroblast Cell Culture

Cultures of healthy control BJ (ATCC^®^ CRL-2522™) fibroblasts were obtained from American Type Culture Collection (ATCC, Manassas, VA, USA) and the ten patient-derived diseased fibroblasts (SBG1, SBG2, SBG3, SBG4, SBG5, SBG6, SBG7, SBG8, SBG9, SBG10) were obtained from the Medical University of Salzburg, Austria. These cells were maintained in a fibroblast expansion medium that consisted of minimal essential medium (MEM) (Thermo Fisher Scientific, Waltham, MA, USA), 10% fetal bovine serum (FBS) (GE Healthcare—HyClone™, Chicago, IL, USA), and 2 mM l-glutamine (Thermo Fisher Scientific). Fibroblasts were enzymatically passaged in 0.05% Trypsin-EDTA (Thermo Fisher Scientific). All cell cultures were maintained without the use of antibiotics, handled in Biosafety Type II sterile hoods regularly cleaned with UV irradiation and 70% ethanol, and grown in 37 °C incubators at 5% CO_2_ and 95% humidity. The culture medium was replenished every two days until cells became 80% confluent. Prior to use in experimentation, cells were dissociated using 0.05% trypsin-EDTA (Thermo Fisher Scientific) and 20,000 cells were seeded into 35mm dishes for fluorescence labeling and image analysis.

### 4.2. Fluorescence Labeling of Mitochondria

To label the mitochondria, fibroblast cells were incubated with MEM NEAA basal medium (Thermo Fisher Scientific) containing 150 nM Mitotracker Red CM-H2Xros (Invitrogen, Waltham, MA, USA) for 30 min. In the FCCP treatment group, fibroblast cells were incubated with 0.7 µM FCCP for 30 min before the addition of the Mitotracker Red CM-H2Xros. At the end of the incubation period, the cells were washed 3 times with prewarmed Dulbecco’s phosphate-buffered saline (dPBS). The nucleus was stained by further incubating cells with basal medium containing Nucblue Hoechst (Invitrogen) for 15 min. Following this incubation, cells were washed several times with prewarmed dPBS to remove excess dye. At the end of the wash, MEM NEAA basal medium was added to each dish prior to image acquisition.

### 4.3. Live-Cell Fluorescence Microscopy

Fluorescence images of live cells were acquired using an EVOS FL inverted light/epifluorescence microscope with 40×/0.65 objective and a Sony ICX445 monochrome CCD digital camera. Red fluorescence from Mitotracker Red CM-H2Xros was measured using a 530 nm excitation and a 593 nm emission filter set. Blue fluorescence from Nucblue Höchst was measured using a 360 nm excitation and a 447 nm emission filter set. All live cells were imaged on 35 mm dishes containing phenol-red-free basal medium. Image acquisition was performed one dish at a time with a maximum time of 30 min between dishes. All dishes were stored in a humidified 37 °C, 5% CO_2_ incubator until image acquisition. All images of live cells were taken on the same day as the labeling of mitochondria. All live-cell images were exported as TIFF files for further analysis. Ten to fourteen images were acquired per dish and three dishes were stained per trial. Three independent trials were performed for each of the fibroblast cell lines used in the study.

### 4.4. MINA Network Analysis

The images generated for the human fibroblast cell lines were preprocessed on ImageJ (NIH, Bethesda, MD, USA) following steps previously outlined [17]. After pre-processing, the images were skeletonized. Post skeletonization, images were segmented using Adobe Photoshop CC 2018. These segmented images were opened in ImageJ, and the MiNA macros were used to quantify mitochondrial morphological parameters of each segmented image. Since fibroblast cell lines have different cellular morphologies, we normalized the parameters generated through MiNA by the cell surface area which was also measured using ImageJ.

### 4.5. Mitochondrial Membrane Potential Measurements

In this study, all fibroblasts were maintained in culture following established protocols until the desired passage (p8) was reached. Prior to use in flow cytometry analysis, cells were cultured until 70–80% confluence was reached. On the day of the experiment, cells were enzymatically detached using 0.05% Trypsin-EDTA (Thermo Fisher Scientific) and centrifuged at 400× *g* for 5 min. Cells were then resuspended in basal medium, after which the desired amount of tetramethylrhodamine, ethyl ester (TMRE- Abcam, Cambridge, MA, USA) was added (for a final concentration of 50 nM). For FCCP and Oligomycin treatment groups, 20 µM and 5 µM of FCCP and Oligomycin were added, respectively for 10 min prior to treatment with TMRE. Cells were incubated with TMRE in a 37 °C, 5% CO_2_ incubator for 25 min. At the end of the incubation period, cells were centrifuged at 400× *g* for 5 min. To wash off the excess dye, cells were resuspended in 1× dPBS solution and centrifuged for another 5 min. At the end of the wash, the cells were resuspended in phenol-red free basal medium and transferred to Accuri C6 plus flow cytometer (BD Biosciences; Franklin Lakes, NJ, USA) for data acquisition. A total of 10,000 events were recorded for each cell line. After data acquisition, the data were exported as FCS files and analyzed using FlowJo_v10.6.2 software (FlowJo LLC, Ashland, OR, USA). To gate for the TMRE-positive population, cells that were not stained with TMRE were used to gate for the TMRE-negative cell populations. Mean fluorescent intensity (MFI) values, a measure of the geometric mean of TMRE-positive cells, were obtained for statistical analysis.

### 4.6. Statistical Analysis

To ensure scientific rigor and reproducibility, an ANOVA design accounting for 3 biological and 10–14 images from healthy control (BJ-fibroblast) and diseased (SBG1, SBG2, SBG3, SBG4, SBG5, SBG6, SBG7, SBG8, SBG9, SBG10) was used to identify any differences with respect to control BJ fibroblasts. Post hoc Tukey HSD test was used to identify differences among specific groups. Data are presented as the mean ± standard deviation (SD) and were analyzed using the GraphPad Prism 8 program (GraphPad Software, San Diego, CA, USA). A *p* < 0.05 was considered significant.

## 5. Conclusions

In summary, we report for the first time the effects of specific mtDNA perturbations (mutations and deletions) on mitochondrial dynamics in the context of specific mitochondrial disorders like LS, KSS, MELAS, and PS. Our results demonstrate that specific point mutations and deletions affecting the OxPhos complexes and translation machinery could upregulate fission dynamics, creating a dysfunction that favors hyperfragmentation of the mitochondria. Although certain mutations and cellular demand cause hyperfused mitochondria, our observations indicate that this is not the predominant morphological adaptation in fibroblast cells with mitochondrial disorders. This study improves our understanding of mitochondrial dynamics in patient fibroblasts with multiple developmental defects and mitochondrial disorders and could lead to a better correlation of mitochondrial dynamics, genetics, and bioenergetics; the specific role of mitochondria in patient diagnosis and prognosis; and focused therapies to treat various mitochondrial disorders.

## Figures and Tables

**Table 1:** Mitochondrial morphology predictions with MiNA descriptors. Mitochondrial fission and fusion cause changes to mitochondrial morphology. The most predominant process dictates mitochondrial morphology and can provide an insight into mitochondrial health.

Mitochondrial Morphology/MiNA Descriptors	Number of Individuals	Number of Networks	Mean Branch Length	Mean Network Size
Fission	Increase	Increase ^1^	Decrease	Decrease
Fusion	Decrease	Decrease ^2^/Increase ^3^	Increase	Increase

^1^ Smaller networks; ^2^ Larger networks form; ^3^ Individuals form networks.

**Table 2:** Mitochondrial morphology summary for fibroblast cell lines. Comparison of mitochondrial morphology of diseased and healthy fibroblast cell lines under basal conditions. Results suggest that diseased cell lines tend to have fewer respiring mitochondria with small branches and fragmented networks. In some cases, however, as seen in SBG5 and SBG7, hyperfusion serves as a compensatory mechanism. * is used to signify data that is statistically significant between healthy and diseased fibroblasts.

Cell Line	Mutation/Deletion	Number of Individuals	Number of Networks	Mean Branch Length	Mean Network Size	Total Respiring Mitochondria
SBG1	*MTATP6* (8993T>G)	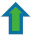	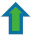	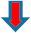 *	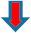	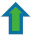
SBG2	*MTATP6* (8993T>G)	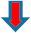	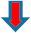	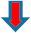 *	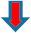	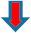
SBG3	*MTATP6* (9185T>C)	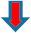	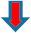 *	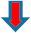	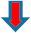	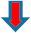
SBG4	*MTND3* (10158T>C)	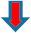	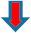 *	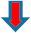	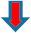	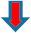
SBG5	*MTND5* (12706T>C)	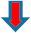 *	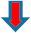 *	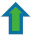 *	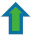 *	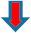 *
SBG6	*MT-TL1* (3243A>G)	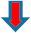 *	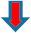 *	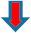 *	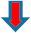 *	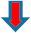 *
SBG7	*MT-TE* (14739G>A)	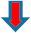 *	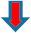 *	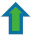	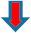 *	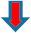 *
SBG8	*MT-Del* (10676∆14868)	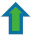	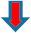	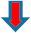 *	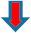	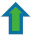
SBG9	*MT-Del* (7342∆9916)	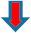	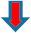	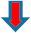	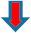	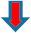
SBG10	*MT-Del* (10167∆15568)	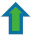	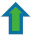	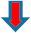 *	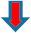	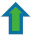

## Data Availability

No dataset has been deposited in a repository, and the data from the studied patient fibroblasts are not publicly available, in agreement with privacy law and our institutional policies.
